# Assessment of Coronal Leakage with Two Intracanal Medicaments After Exposure to Human Saliva-An *In Vitro* Study

**DOI:** 10.5005/jp-journals-10005-1548

**Published:** 2018-10-01

**Authors:** Selva Balaji, Krishna Kumar, Ramesh Venkatesan, Shantham Krishnamoorthy, Vidya Manoharan, Senthilkumar Marimuthu

**Affiliations:** 1 Reader, Department of Pedodontics and Preventive Dentistry, Indira Gandhi Institute of Dental Sciences, Sri Balaji Vidyapeeth, Puducherry, India; 2 Professor, Department of Pedodontics and Preventive Dentistry, Rajah Muthiah Dental College, Tamil Nadu, India; 3 Senior Lecturer, Department of Pedodontics and Preventive Dentistry, Indira Gandhi Institute of Dental Sciences, Sri Balaji Vidyapeeth, Puducherry, India; 4 Senior Lecturer, Department of Pedodontics and Preventive Dentistry, Sathyabama Dental College, Tamil Nadu, India; 5 Senior Lecturer, Department of Pedodontics and Preventive Dentistry, Royal Dental College, Kerala, India; 6 Senior Lecturer, Department of Conservatives and Endodontics, Madha Dental College, Tamil Nadu, India

**Keywords:** Calcium hydroxide, Chlorhexidine digluconate, Dental leakage, Root canal therapy, Temporary dental restorations.

## Abstract

**Introduction:**

One among the various reasons for root canal failure in endodontics is the leakage of an intracanal medicament due to improper coronal sealing.

**Aim:**

To assess the coronal leakage of two intracanal medicaments sealed with two different temporary filling materials.

**Materials and methods:**

An *in-vitro* study was done on 55 teeth where they were divided into three groups with two root canal medicaments namely calcium hydroxide + 0.2% chlorhexidine solution, triple antibiotic paste, and a control group. These three groups were restored temporarily with MD Temp and IRM, and these samples were checked for coronal leakage after 30 days.

**Results:**

The group, triple antibiotic paste with IRM stayed for more number of days without leakage with the mean of 24.5 days, followed by group triple antibiotic paste with MD Temp. The least coronal leakage was seen in group MD Temp without Intracanal medicament with the mean of eight and half days followed by group IRM without Intracanal medicament. When comparing the two temporary filling materials without any medicament, there was no significant difference between the same. When comparing within MD Temp group, the least microleakage was seen with Triple antibiotic paste with MD Temp. In the IRM group, the least microleakage was seen with Triple antibiotic paste with IRM.

**Conclusion:**

Triple antibiotic paste was found to be the most promising intracanal medicament with an appropriate seal.

**Clinical significance:**

The best intracanal medicament, which is triple antibiotic paste in the present study could prevent microorganism leakage and inhibit bacterial growth.

**How to cite this article:** Balaji S, Kumar K, Venkatesan R, Krishnamoorthy S, Manoharan V, Marimuthu S. Assessment of Coronal Leakage with Two Intracanal Medicaments After Exposure to Human Saliva-An *In Vitro* Study. Int J Clin Pediatr Dent., 2018;11(5):406-411.

## INTRODUCTION

Root canal treatment aims at preventing the entry of organisms into the canal system or eliminating them if already present. Subsequently, a hermetic seal is placed to prevent the bacterial invasion at the apical region.^1^ The by-products of bacteria cause inflammation at the apex.^2^

The properties of the ideal coronal filling material are that it should provide better sealing of tooth margins, lack porosity and withstand dimensional changes to hot and cold temperatures. It should be easily inserted and removal, good abrasion, and compression resistance, compatible with root canal medicaments, and provide better esthetics.^3^ In case of loss of coroal filling, there will be periapical inflammation which could result in the failure of endodontic treatment.^4^ Leakage of coronal seal is observed to be etiology of root canal failure.^5^

Intracanal medicament should possess an extensive antibacterial activity should have properties that allows the medicament to pass through the tubules and lateral canals of the root canal.^6^ The commonly used intracanal medicaments are calcium hydroxide, ledermix paste, chlorhexidine gluconate gel, triple antibiotic paste, propolis, etc.^7^

Calcium hydroxide (CH) has hydroxyl ions exhibiting antimicrobial characteristics. It has a high pH of 12 to 12.5, which destroys the cell wall membrane of bacteria and also the synthesis of protein by bacteria. Chlorhexidine digluconate (CHX) has wide spectrum antimicrobial activity with prolonged action. The combination of CH and CHX when used as root canal medicine has shown promising results.^8^

A triple antibiotic paste containing ciprofloxacin, metronidazole, and minocycline is being used for lesion sterilization and tissue repair. This 3 mix paste was found to be effective in persistent endodontic infections.^9^ Hence, the study aimed to assess time needed for the recontami-nation of root canals treated with two intracanal medicaments (Ca(OH)_2_ + 0.2% Chlorhexidine solution and triple antibiotic paste followed by sealing with two temporary materials namely MD Temp and IRM coronally)

## MATERIALS AND METHODS

Sixty caries-free teeth with single straight root were taken for the present in-vitro study. The study was conducted at the Department of Pedodontics and Preventive Dentistry at Rajah Muthiah Dental College & Hospital, Tamil Nadu. (RMDCH), TN. Conventional access was prepared in fifty-five teeth. Uniform access was made in 55 teeth with no # 4 round bur. The specimens were then flared in the coronal portion with a Gates-Glidden of size 4 (Mani, Japan), followed by size three and two. Later Size 10 K-file (Mani, Japan) was introduced into each canal until it reaches the apex. The measurement of the working length was confirmed by subtracting 1 mm from apex. The canal was recapitulated with same K-file and the patency of the canal was preserved.

K-files with step back technique did preparation of root canals. Enlargement of apical foramen up to size 20 K-file was done to standardize the diameter. The preparation was done up to a size of 40 K-file. one ml of 2.5% NaOCl (sodium hypochlorite) solution was used to irrigate the canal between files. To remove the smear layer, 17% EDTA was used for 3 minutes and then irrigated with 1 mL of 2.5% NaOCl. A quantity 5mL of sterile saline did the final rinse, thereby the debris and remaining irrigant was removed. Absorbent points were used to dry the canals before placing intracanal medicament.

The teeth were randomly divided into groups as follows:

*Group I:* MD Temp

(IA) 5 teeth without intracanal medicament.

(IB) 10 teeth with Ca(OH)2 + 0.2% Chlorhexidine solution.

(IC) 10 teeth with triple antibiotic paste.

*Group II:* I.R.M

(II A) 5 teeth without intracanal medicament.

(II B) 10 teeth with Ca(OH)2 + 0.2% Chlorhexidine solution.

(II C) 10 teeth with triple antibiotic paste.

Group III: 5 teeth is coronally unsealed

The apparatus to evaluate leakage used in our study was done as per methods mentioned by Siqueira et al.^10^ Round bur (no. 2) and high-speed handpiece was used to make a hole in the center of the rubber stopper in which each tooth was inserted under pressure up to its cementoe-namel junction (CEJ), so that crown portion of the tooth was outside the vial and its root was within glass vial.

Each tooth, stopper, and vials were autoclaved and then adapted. Vials were then filled with (Brain Heart Infusion) BHI broth.

### Medicaments Preparation

Calcium hydroxide (60 mg) mixed with 100 ml of 0.2% chlorhexidine solution was placed into the canal with a size 35 lentulo spiral as mentioned by Farhad et al. in 2012.^8^

Preweighed medicaments ciprofloxacin, metronida-zole and minocycline (250 mg each) were mixed with sterile water to make a triple antibiotic paste. The medication was introduced into the canal using 20 gauge needle which was placed 2 mm short of the WL to the level of (CEJ) by backfill approach as described earlier by Reynolds et al. in 2009.^11^

The teeth were sealed with a small pellet of cotton and two different temporary filling materials as mentioned in the groups, respectively.

Cylinders were prepared by cutting the tip of the 20 mL plastic syringes. A chamber was created around the crown of the teeth by adapting the cylinders to the outer surface of the stoppers. Silicone sealant was applied between the tooth and the stopper and also between the flask and the stopper to avoid penetration of saliva into the BHI broth.

Unstimulated saliva (20 mL) was collected from an individual who volunteered for every 3 days at 9 AM. The individual was instructed not to use tooth cleaning aids for 12 hours before saliva collection. Human saliva and BHI broth were filled in each apparatus with the proportion of 1:3 ratio. The crown of the teeth was exposed to the saliva inside the cylinder for 30 days. The apparatus was incubated at 37° C. Saliva and BHI broth was replaced once in three days. The time of appearance of turbidity in the vials was noted visually by a single examiner on a daily basis for a month. This showed that microleakage occurred.

An analysis was done using the Statistical Package for Social Sciences 19 (SPSS Chicago link). The obtained data were tabulated and analyzed using an independent t-test for comparison of the mean number of days without leakage, between respective groups.

## RESULTS

Table 1 shows the inter-comparison of group IA (MD Temp without Intracanal Medicament) with the mean number of days as 7.50 + 0.71 compared to IIA (I.R.M without intracanal medicament) 8.50 + 0.71 showed the statistically non-significant difference between the groups since p-value was 0.29 (> 0.05). This showed that when comparing group IA and IB there was no difference in the leakage in both groups.

Table 2 shows the comparison within the MD Temp groups IA (MD temp without intracanal medicament) with the mean number of days as 7.50 + 0.71, group IB (MD temp with Ca(OH)_2_ + 0.2% CHX) with the mean number of days as 13.50 + 1.29, group IC (MD Temp + Triple antibiotic paste) with the mean number of days as 23.00 + 1.83.group. All the groups showed a significant difference with a p = 0.001 (< 0.05). This showed that when there is inter-comparison of group IA, IB and IC, group IC stayed more number of days without leakage followed by group IB, whereas group IA stayed the least number of days without leakage.

Table 3 shows the comparison within the IRM groups IIA (IRM without intracanal medicament) with the mean number of days as 8.50 + 0.71, IIB (IRM with (Ca(OH)2 + 0.2% CHX) with the mean number of days as 17.40 + 3.65, IIC (IRM + Triple antibiotic paste) with the mean number of days as 24.50 + 2.65 and II B (I RM + (Ca(OH)_2_ +0.2% CHX) *vs.* IIC (I RM + Triple antibiotic paste). All the groups showed a statistically significant difference with a p-value of 0.001 (< 0.05). Thus when inter-comparison of groups were done between Groups II A , II B, and II C, Group II C stayed more number of days without leakage followed by II B, while least number of days without leakage was observed in group IIA.

**Fig. 1:**
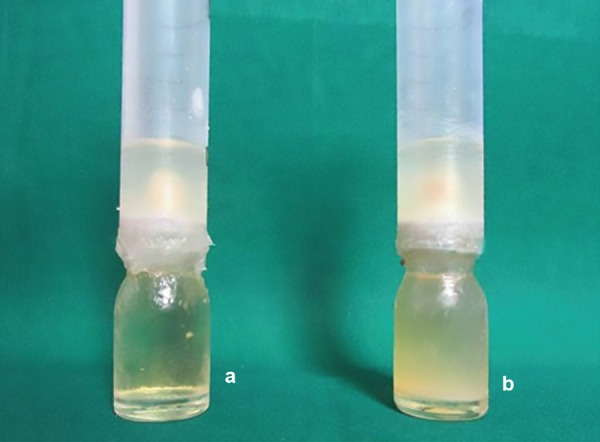
Formation of turbidity, 4a-Before turbidity, 4b-After turbidity

Figure 1 shows the difference in the formation of turbidity

**Graph.1:**
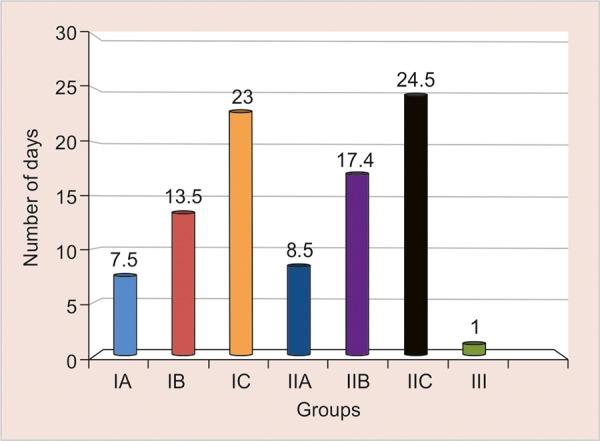
Mean (number of days) coronal leakage of all the group.

Graph 1 shows the comparison of groups IA (MD temp + (Triple Antibiotic paste), IB (MD Temp with Ca(OH)_2_ + 0.2% CHX), IC (MD Temp + Triple antibiotic paste), II-A (IRM without intracanal medicament), IIB (IRM with (Ca(OH)_2_ + 0.2% CHX), IIC (IRM + Triple antibiotic paste), III (5 teeth is coronally unsealed). In this group IIC stayed more number of days without leakage with the mean of 24.5 days, followed by group IC with 23 days, and group IIB with 17.4 days, followed by group IB with 13.5 days, Least number of days was observed with Group IIA with 8.5 days, followed by group IA with 7.5 days and group III with 1 day. Thus triple antibiotic paste with IRM withstood coronal leakage for a longer time, preventing recontamination.

**Table 1:** **Table. 1:** Comparison of the mean number of days without leakage between groups without intracanal medicament

*Group*		*Samples*		*Mean (days)*		*Std. Deviation*		*t-value*		p-value*	
IA *vs* IIA		MDTemp without intracanal medicament		7.50		0.71		1.41		0.29 Not significant	
		IRM without intracanal medicament		8.50		0.71					

*Independent t-test is used for comparison of the groups

**Table 2:** **Table. 2:** Comparison of the mean number of days without leakage between MD Temp groups

*Group*		*Samples*		*Mean (days)*		*Std. Deviation*		*t-value*		p-value*	
IA *vs* IIA		MD Temp without intracanal medicament		7.50		0.71		5.90		0.001 Significant	
		MD Temp with (Ca(OH)_2_ + 0.2% CHX)		13.50		1.29					
IA *vs* IC		MD Temp without intracanal medicament		7.50		0.71		11.04		0.29 Not Significant	
		MD Temp + Triple antibiotic paste		23.00		1.83					
I B *vs* II C		MD Temp + (Ca(OH)_2_ + 0.2% CHX)		13.50		1.29		8.49		0.29 Not Significant	
		CHX) MD Temp + Triple antibiotic paste		23.00		1.83					

*Independent t-test is used for comparison of the groups

**Table 3:** **Table 3:** Comparison of the mean number of days without leakage between IRM groups

*Group*		*Samples*		*Mean (days)*		*Std. deviation*		*t-value*		p-value*	
IIA *vs* IIB		IRM without intracanal medicament		8.50		0.71		3.24		0.02 Significant	
		IRM with (Ca(OH)2 + 0.2% CHX)		17.40		3.65					
IIA *vs* IIC		IRM without intracanal medicament		8.50		0.71		7.96		0.001 Significant	
		IRM + Triple antibiotic paste		24.50		2.65					
IIB *vs* IIC		IRM + (Ca(OH)_2_ + 0.2% CHX)		17.40		3.65		3.25		0.001 Significant	
		IRM + Triple antibiotic paste		24.50		2.65					

*Independent t-test is used for comparison of the groups

## DISCUSSION

Penetration of bacterial products may initiate the inflammation, and leakage of saliva may initiate the growth of bacteria inside the root canals.^12^ To test the sealing ability of endodontic materials, various leakage studies have been done using cultures and saliva, which are considered to be more relevant both biologically and clinically than dye leakage test which was used traditionally. Since the molecular weight of the dye was low they can reach to the sites were bacterial cells cannot. Thus, saliva leakage test closely mimics the real clinical scenario. The advantages of human saliva over bacterial cultures are the presence of numerous and various bacterial species, high density of bacteria (-10s bacterial cells/mL) and their by-products. Enzymes, proteins, and other elements of natural saliva not given by culture media.^13^

The success of noninvasive root canal treatment could be compromised by Coronal leakage. Therefore, a tempo-rary restorative material should prevent marginal leakage in between appointments and before a final coronal seal is placed.^14^

Though temporary filling materials reduce microorganism leakage to an extent, root canal medicaments also act to prevent entry of oral microorganisms into the canal system in the event of a breakdown of the interim filling. To prevent root canal contamination, a right amount of root canal medicament is needed.^15^

Root canal medicaments may prevent bacterial penetration in two ways. First due to the antibacterial properties which act as a chemical barrier against leakage by killing bacteria, thus preventing their way into the root canal. Second, by creating a physical barrier for entry of bacteria.^13^

Several benefits of Ca(OH)_2_ have been proposed when used as a root canal medication during root canal treatment. It is well documented that Calcium hydroxide is a root canal medicament which can be mainly attributed to its antimicrobial property. Calcium hydroxide causes alteration of the bacterial cell wall and denatures lipopolysaccharides.^16^

Chlorhexidine (CHX) has a wide range of action against all kinds of bacteria and Candida species. CHX was more effective compared to Ca(OH)_2_ when it was used as an intracanal medicament in eliminating *E. faecalis* from inside dentinal tubules.^17^

Systemic administration is not so effective compared to the local application of antibiotics in root canal infection. It could be due to its diverse microflora and complex nature. Also, a single antibiotic might not cause effective disinfection of the root canal system.^18^ Thus, Triple antibiotic paste (TAP) as an intracanal medicament, could prevent microorganism leakage and inhibit bacterial growth. None of the studies have reported about coronal leakage when using TAP as an intracanal medicament; therefore TAP was used as another root canal medicament. Coronal leakage was considered as a potential causative factor in the failure of RCTs.^19^ Therefore the inter appointment visit during the RCT should be less than two weeks to prevent recontamination.

The present study showed that placement of a temporary filling material had minimal resistance to microleak-age. This was in agreement to a recently performed study by Udayakumar et al. where he concluded that all coronal restorations failed to prevent microleakage beyond a week.^14^ However the intracanal medication is of great significance as it not only act as a barrier but also inhibits bacterial growth for some period of time. This was supported by research done by Deveaux et al. (1999)^20^ and Aledrissy et al. (2011)^21^ who found that the inconsistency in the mixing process and resulting lack of homogeneity lowers its sealability and explains the increase in leakage. Because of these zinc oxide, eugenol based materials are less leak-proof among temporary restorative materials. A recent study compared Cavit (Zinc oxide base), IRM (Zinc oxide eugenol base) and CLIP (Methacrylate base) using the dye penetration methods and concluded that Clip (Hydroethylmethacrylate, butylhydroxytoluene, acrylate esters, polymers-Voco, Cuxhaven, Germany) exhibited the least microleakage followed by IRM and Cavit.^22^

In the present study, triple antibiotic paste with MD Temp and IRM had the least microleakage when compared to Ca(OH)_2_ + 0.2% CHX. Which was similar to Sato et al. (1996)^19^ who accessed the potential of TAP to destroy bacteria in the deeper part of root canal dentin and In 24 hrs after applying TAP no bacteria was found in the infected dentin in root canals of most of the teeth.

According to Thu et al. (2013)^23^ MD Temp showed less microleakage when compared to caviton and zinc oxide eugenol. IRM is a form of reinforced ZOE material. To make the material relatively hydrophobic, polymethyl-methacrylate has been added to maintain its integrity in aqueous solutions.^13^

In our study the comparison of temporary filling, materials did not show any significant difference. Therefore they don’t play an important role in preventing microleakage.

According to Farhad et al.,^8^ distilled water/Calcium hydroxide combination has less antibacterial activity when compared to chlorhexidine/ Calcium hydroxide combination as a root canal medicament. Therefore in this study Ca(OH)_2_ + 0.2% chlorhexidine was used a root canal medicament.

In 7 days, CHX inhibited *E. Faecalis* colonization in bovine roots as a root canal medicament. This showed that CHX exhibits antimicrobial substantivity in root dentin.^24^

Usually in the sterilization of root canals and healing of periapical pathology TAP has been used. Discoloration of tooth and formation of resistant bacterial strains are the major drawbacks of the TAP. It is favorable in sterilization and revascularization of the pulpal tissue.^25^

## CONCLUSION

Hence, from the above study triple antibiotic paste was most promising and could be used as an intracanal medicament with an appropriate coronal seal. However, when the temporary filling materials were compared within them, there was no statistical significant difference. Further in vivo studies with more samples and with more parameters replicating exact oral condition were needed.

## CLINICAL SIGNIFANCE

The best intracanal medicament, which is triple antibiotic paste in the present study could prevent microorganism leakage and inhibit bacterial growth than calcium hydroxide with chlorhexidine.
